# Supplementary material for: Analysis and control of untemplated DNA polymerase activity for guided synthesis of kilobase-scale DNA sequences
Source: Nat Commun. 2026 Feb 26;17:3251. doi: 10.1038/s41467-026-69915-x (PMC13061963; doi:10.1038/s41467-026-69915-x)
Supplement: Supplementary file 1 — Supplementary Information [file 41467_2026_69915_MOESM1_ESM.pdf]

Supplementary Information for:

Analysis and control of untemplated DNA polymerase activity for guided synthesis of kilobase-scale DNA sequences

Simeon D. Castle, Thea C.T. Irvine, Adrian Woolfson, Gregory Linshiz, Blake T. Riley, Ifor D.W. Samuel, Loren Picco, Philipp Holliger, Lauren M. Oldfield, Andrew Hessel, and Thomas E. Gorochowski

Supplementary Figures		Page
Supplementary Figure 1:	Sequence analysis of Taq (65°C, Taq Standard Buffer)	2
Supplementary Figure 2:	Sequence analysis of Taq (74°C, Taq Standard Buffer)	3
Supplementary Figure 3:	Sequence analysis of Vent (65°C, Thermopol Buffer)	4
Supplementary Figure 4:	Sequence analysis of Vent (74°C, Thermopol Buffer)	5
Supplementary Figure 5:	Distributions of read lengths for barcoded and high-molecular weight nanopore sequencing protocols	6
Supplementary Figure 6:	Sequence analysis of Taq (65°C, Minimal pH 8.2 Buffer)	7
Supplementary Figure 7:	Sequence analysis of Taq (65°C, Minimal pH 9.5 Buffer)	8
Supplementary Figure 8:	Sequence analysis of Taq (65°C, Taq Standard Buffer, 25 mM $MgCl_2$)	9
Supplementary Figure 9:	Sequence analysis of Taq (74°C, Minimal pH 8.2 Buffer)	10
Supplementary Figure 10:	Single-molecule analysis of the effect of thermocycling on doodling activity of Taq and Vent polymerases	11
Supplementary Figure 11:	Sequence analysis of Vent exo– (65°C, Thermopol Buffer)	12
Supplementary Figure 12:	Sequence analysis of Therminator (65°C, Thermopol Buffer)	13
Supplementary Figure 13:	Sequence analysis of RT521K (65°C, Thermopol Buffer, 25 mM $MnCl_2$)	14
Supplementary Figure 14:	Sequence analysis of 3A10 (65°C, Taq Standard Buffer, 25 mM $MgCl_2$)	15
Supplementary Figure 15:	Sequence analysis of Taq (65°C, Taq Standard Buffer, only adenine and thymine provided	16
Supplementary Figure 16:	Nanopore sequencing of inosine bases	17
**Supplementary Tables**		
Supplementary Table 1:	Doodling activity of Taq with differing dNTP availability	18

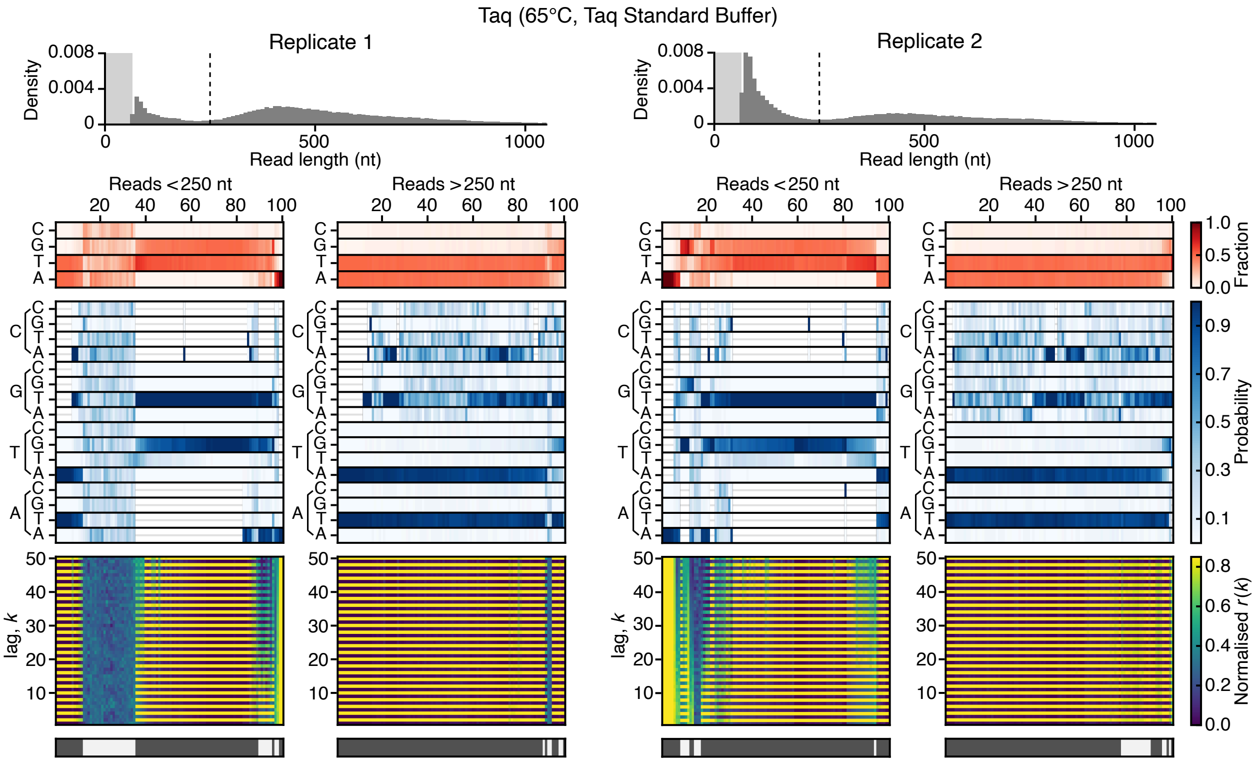

Supplementary Figure 1: Sequence analysis of Taq (65°C, Taq Standard Buffer). The top histograms show the sequence length distribution with the lightly shaded region denoting the 0–65 nt range and dashed line denoting the 250 nt read length. Below this, heatmaps show for a random subset of reads smaller and larger than 250 nt (left and right plots, respectively) the following information (top–bottom): 1. Sequence composition (red heatmap), 2. Probability of transitioning from one base to another (blue heatmap), 3. Autocorrelation analysis capturing the similarity of the sequence compared to itself after varying nucleotide shifts/lag *k* (blue to yellow heatmap), and 4. the seven top clusters of reads (alternating dark and light grey). Reads are displayed vertically and hierarchically clustered such that similar sequences are grouped.

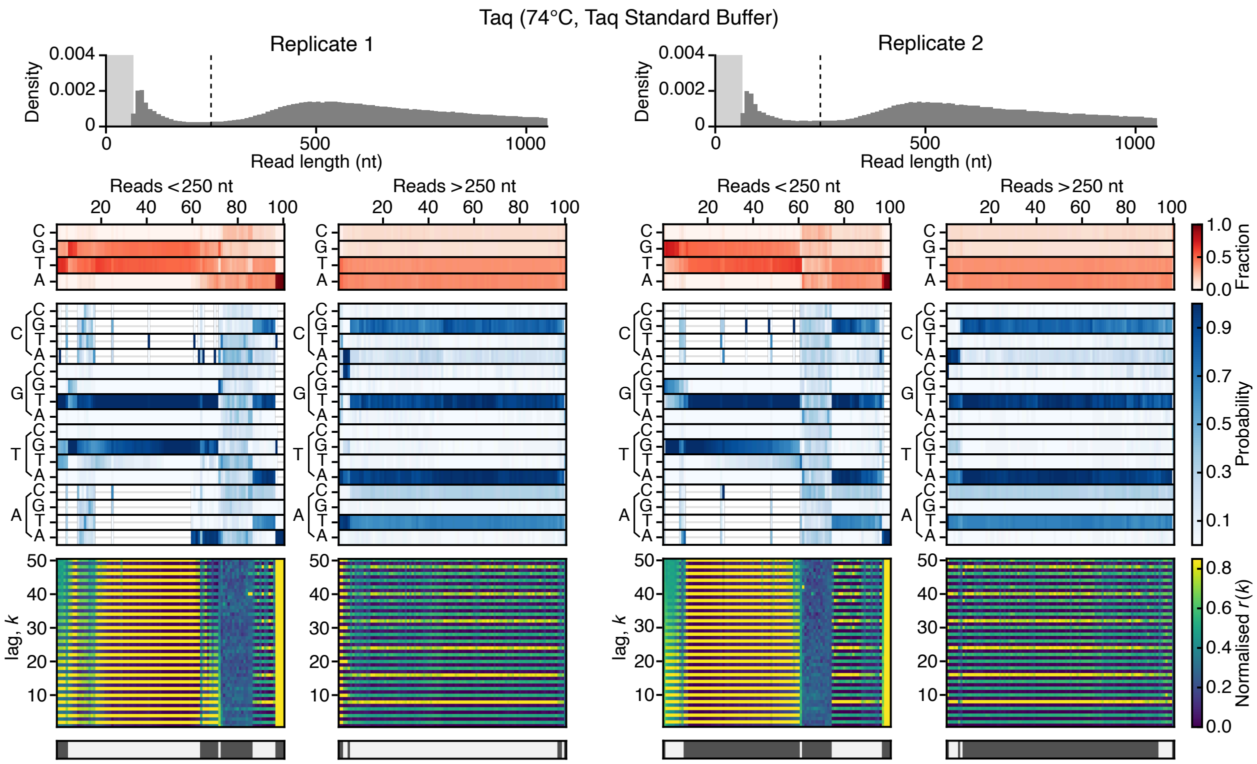

Supplementary Figure 2: Sequence analysis of Taq (74°C, Taq Standard Buffer). The top histograms show the sequence length distribution with the lightly shaded region denoting the 0–65 nt range and dashed line denoting the 250 nt read length. Below this, heatmaps show for a random subset of reads smaller and larger than 250 nt (left and right plots, respectively) the following information (top–bottom): 1. Sequence composition (red heatmap), 2. Probability of transitioning from one base to another (blue heatmap), 3. Autocorrelation analysis capturing the similarity of the sequence compared to itself after varying nucleotide shifts/lag k (blue to yellow heatmap), and 4. the seven top clusters of reads (alternating dark and light grey). Reads are displayed vertically and hierarchically clustered such that similar sequences are grouped.

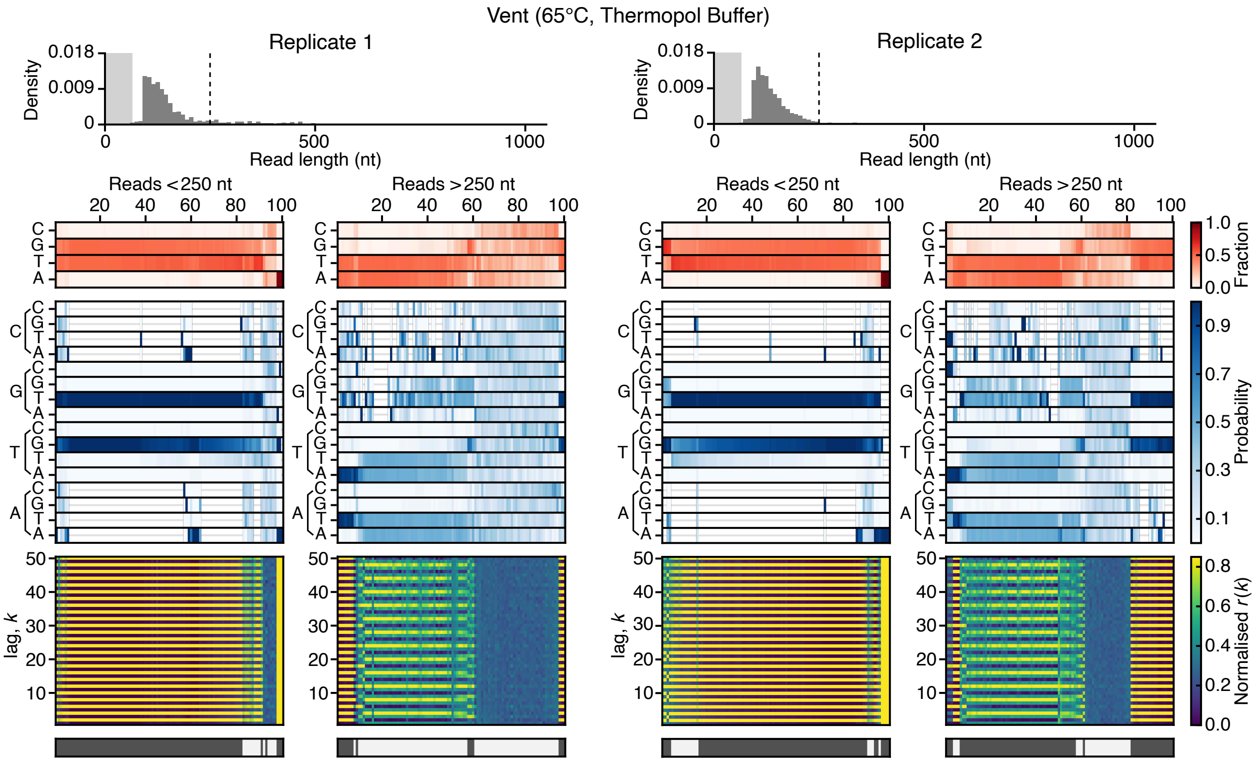

Supplementary Figure 3: Sequence analysis of Vent (65°C, Thermopol Buffer). The top histograms show the sequence length distribution with the lightly shaded region denoting the 0–65 nt range and dashed line denoting the 250 nt read length. Below this, heatmaps show for a random subset of reads smaller and larger than 250 nt (left and right plots, respectively) the following information (top–bottom): 1. Sequence composition (red heatmap), 2. Probability of transitioning from one base to another (blue heatmap), 3. Autocorrelation analysis capturing the similarity of the sequence compared to itself after varying nucleotide shifts/lag k (blue to yellow heatmap), and 4. the seven top clusters of reads (alternating dark and light grey). Reads are displayed vertically and hierarchically clustered such that similar sequences are grouped.

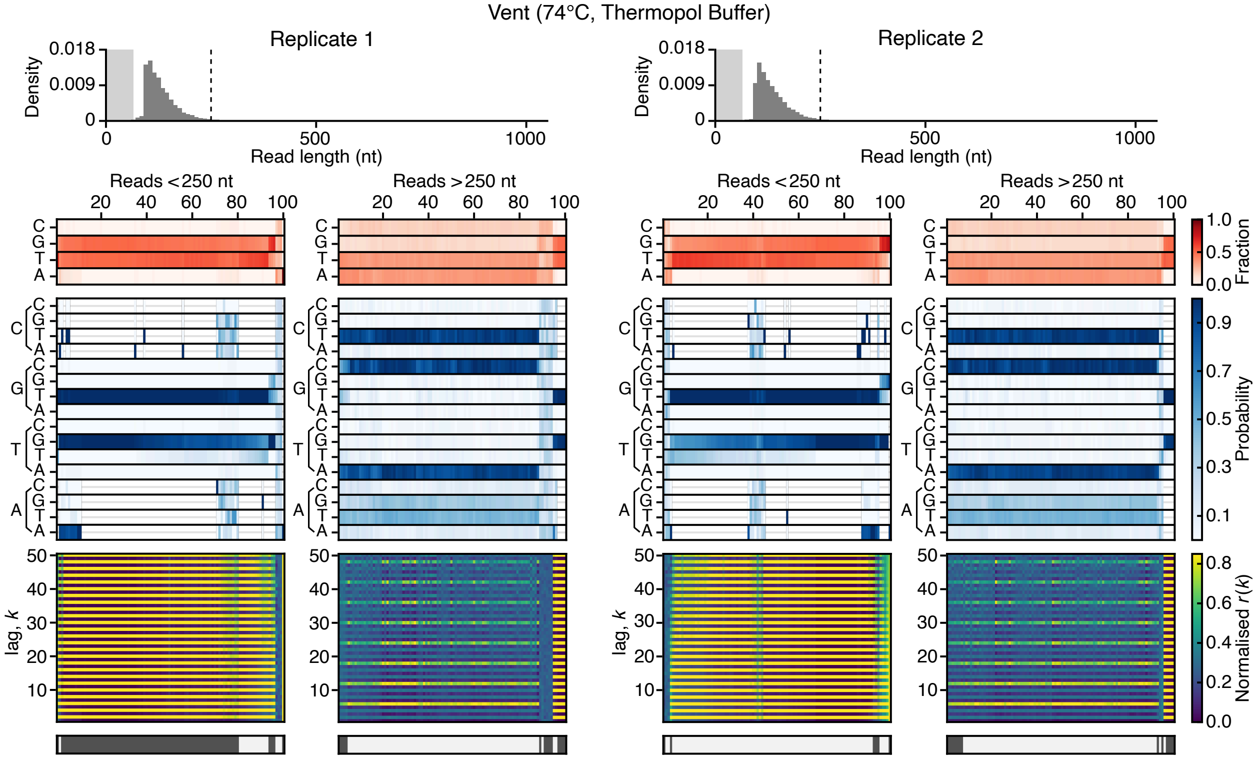

Supplementary Figure 4: Sequence analysis of Vent (74°C, Thermopol Buffer). The top histograms show the sequence length distribution with the lightly shaded region denoting the 0–65 nt range and dashed line denoting the 250 nt read length. Below this, heatmaps show for a random subset of reads smaller and larger than 250 nt (left and right plots, respectively) the following information (top–bottom): 1. Sequence composition (red heatmap), 2. Probability of transitioning from one base to another (blue heatmap), 3. Autocorrelation analysis capturing the similarity of the sequence compared to itself after varying nucleotide shifts/lag *k* (blue to yellow heatmap), and 4. the seven top clusters of reads (alternating dark and light grey). Reads are displayed vertically and hierarchically clustered such that similar sequences are grouped.

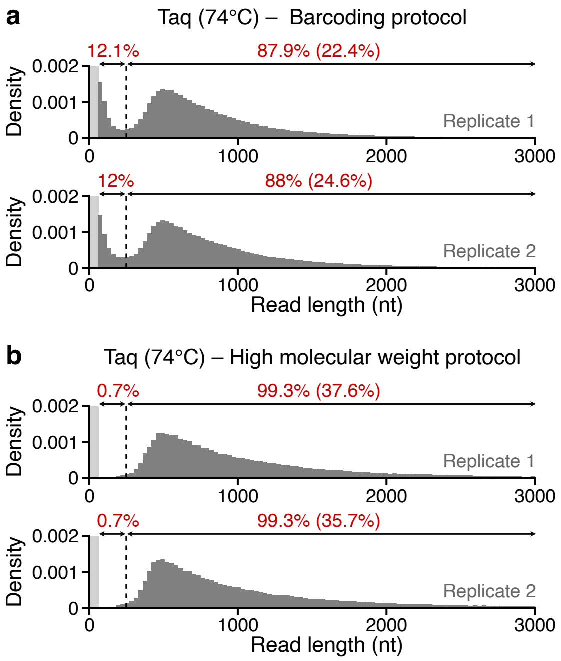

Supplementary Figure 5: Distributions of read lengths for barcoded and high-molecular weight nanopore sequencing protocols. Doodling reactions performed with Taq DNA polymerase at 74°C. (**a**) Samples prepared using barcoding protocol. (**b**) Samples prepared using high molecular weight protocol. Light grey shaded regions denotes reads <65 nt that were filtered from the analysis. Vertical dashed lines denote a read length of 250 nt. Percentages in red show the fractions of reads 65–250 nt (left) and >250 nt long (right), while the value in parenthesis shows the fraction of reads >1,000 nt long.

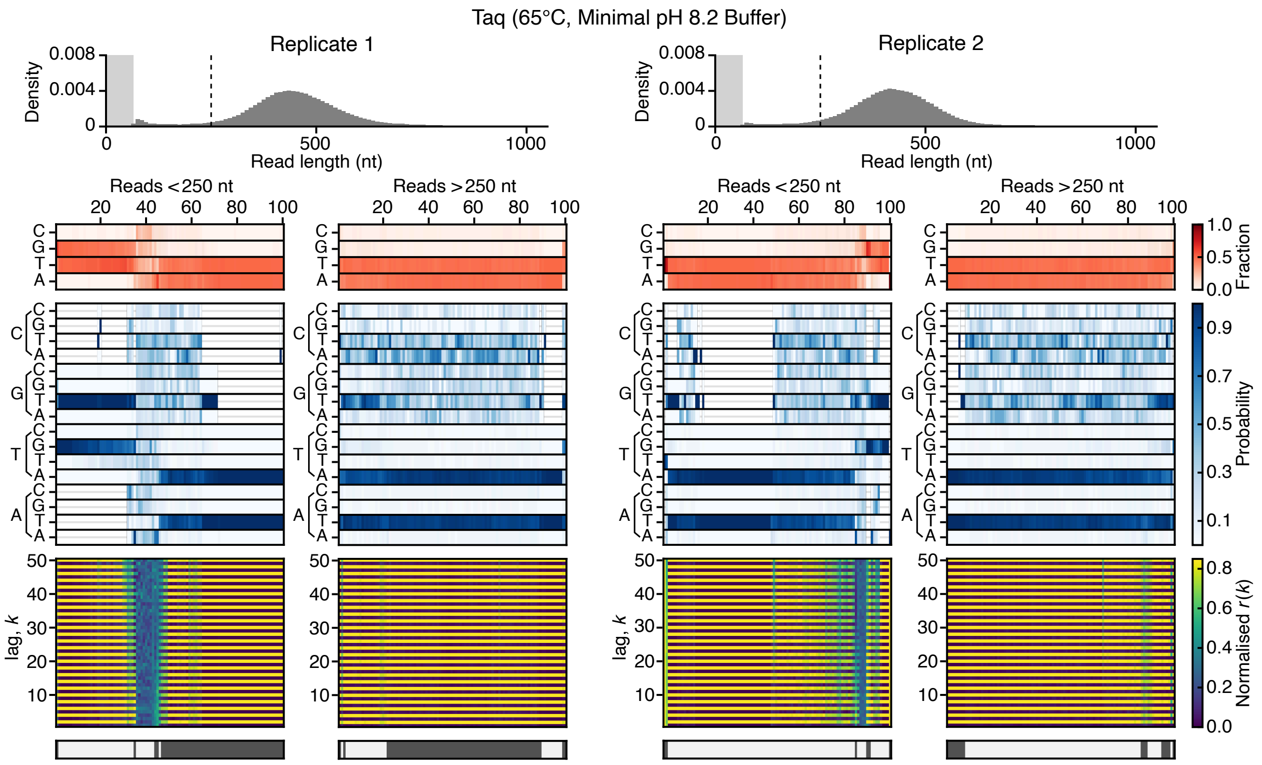

Supplementary Figure 6: Sequence analysis of Taq (65°C, Minimal pH 8.2 Buffer). The top histograms show the sequence length distribution with the lightly shaded region denoting the 0–65 nt range and dashed line denoting the 250 nt read length. Below this, heatmaps show for a random subset of reads smaller and larger than 250 nt (left and right plots, respectively) the following information (top–bottom): 1. Sequence composition (red heatmap), 2. Probability of transitioning from one base to another (blue heatmap), 3. Autocorrelation analysis capturing the similarity of the sequence compared to itself after varying nucleotide shifts/lag *k* (blue to yellow heatmap), and 4. the seven top clusters of reads (alternating dark and light grey). Reads are displayed vertically and hierarchically clustered such that similar sequences are grouped.

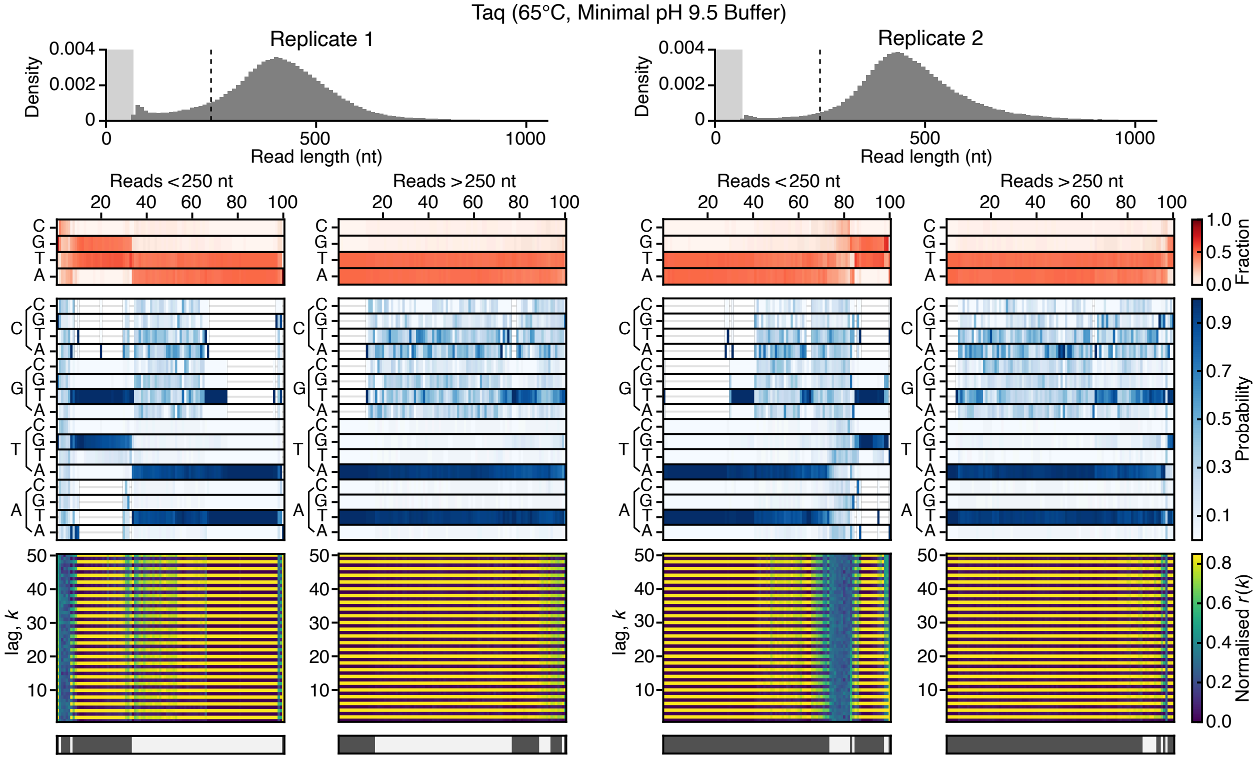

Supplementary Figure 7: Sequence analysis of Taq (65°C, Minimal pH 9.5 Buffer). The top histograms show the sequence length distribution with the lightly shaded region denoting the 0–65 nt range and dashed line denoting the 250 nt read length. Below this, heatmaps show for a random subset of reads smaller and larger than 250 nt (left and right plots, respectively) the following information (top–bottom): 1. Sequence composition (red heatmap), 2. Probability of transitioning from one base to another (blue heatmap), 3. Autocorrelation analysis capturing the similarity of the sequence compared to itself after varying nucleotide shifts/lag *k* (blue to yellow heatmap), and 4. the seven top clusters of reads (alternating dark and light grey). Reads are displayed vertically and hierarchically clustered such that similar sequences are grouped.

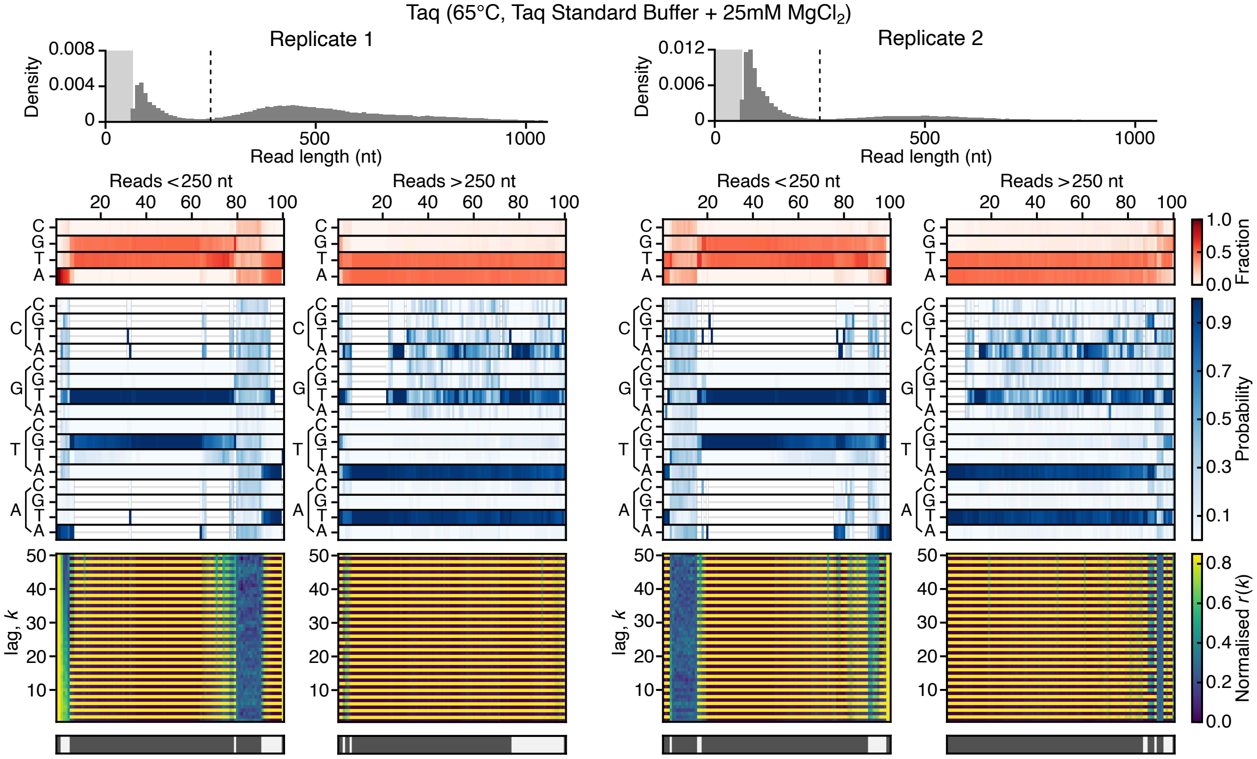

Supplementary Figure 8: Sequence analysis of Taq (65°C, Taq Standard Buffer, 25 mM $MgCl_2$). The top histograms show the sequence length distribution with the lightly shaded region denoting the 0–65 nt range and dashed line denoting the 250 nt read length. Below this, heatmaps show for a random subset of reads smaller and larger than 250 nt (left and right plots, respectively) the following information (top–bottom): 1. Sequence composition (red heatmap), 2. Probability of transitioning from one base to another (blue heatmap), 3. Autocorrelation analysis capturing the similarity of the sequence compared to itself after varying nucleotide shifts/lag *k* (blue to yellow heatmap), and 4. the seven top clusters of reads (alternating dark and light grey). Reads are displayed vertically and hierarchically clustered such that similar sequences are grouped.

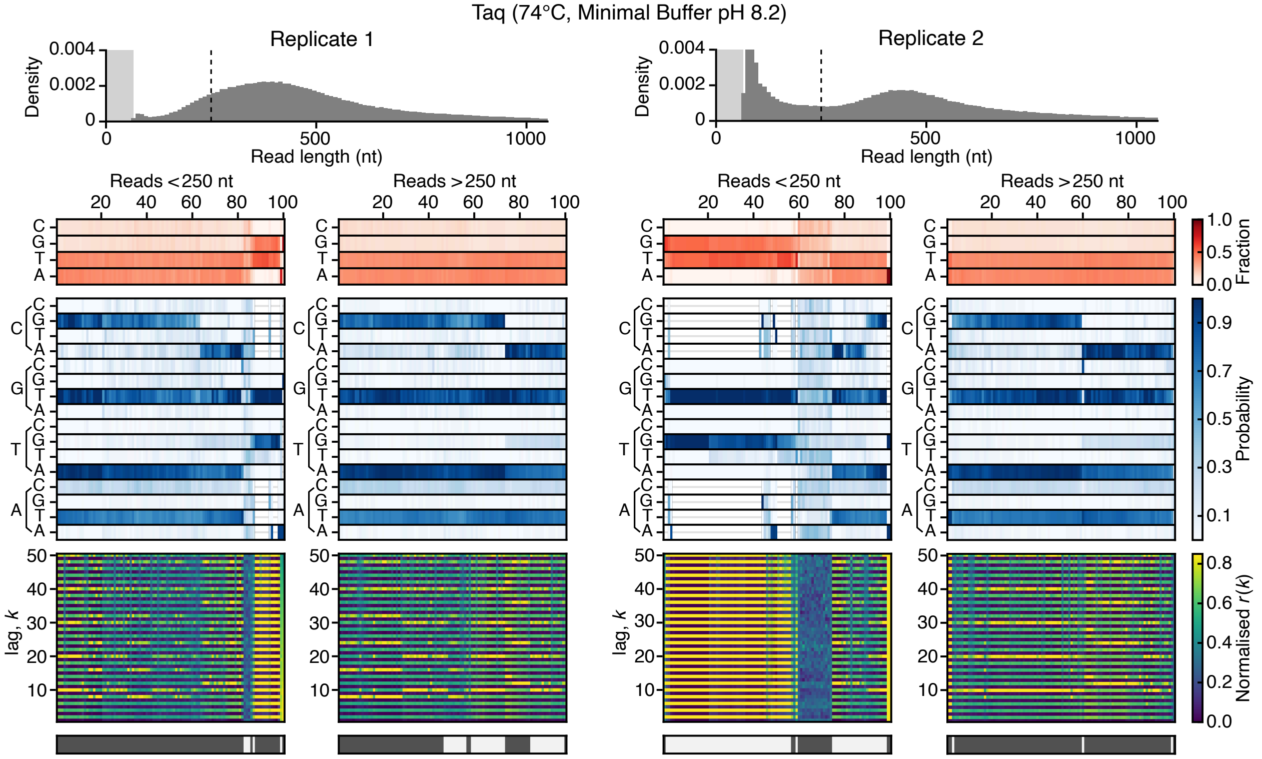

Supplementary Figure 9: Sequence analysis of Taq (74°C, Minimal pH 8.2 Buffer). The top histograms show the sequence length distribution with the lightly shaded region denoting the 0–65 nt range and dashed line denoting the 250 nt read length. Below this, heatmaps show for a random subset of reads smaller and larger than 250 nt (left and right plots, respectively) the following information (top–bottom): 1. Sequence composition (red heatmap), 2. Probability of transitioning from one base to another (blue heatmap), 3. Autocorrelation analysis capturing the similarity of the sequence compared to itself after varying nucleotide shifts/lag *k* (blue to yellow heatmap), and 4. the seven top clusters of reads (alternating dark and light grey). Reads are displayed vertically and hierarchically clustered such that similar sequences are grouped.

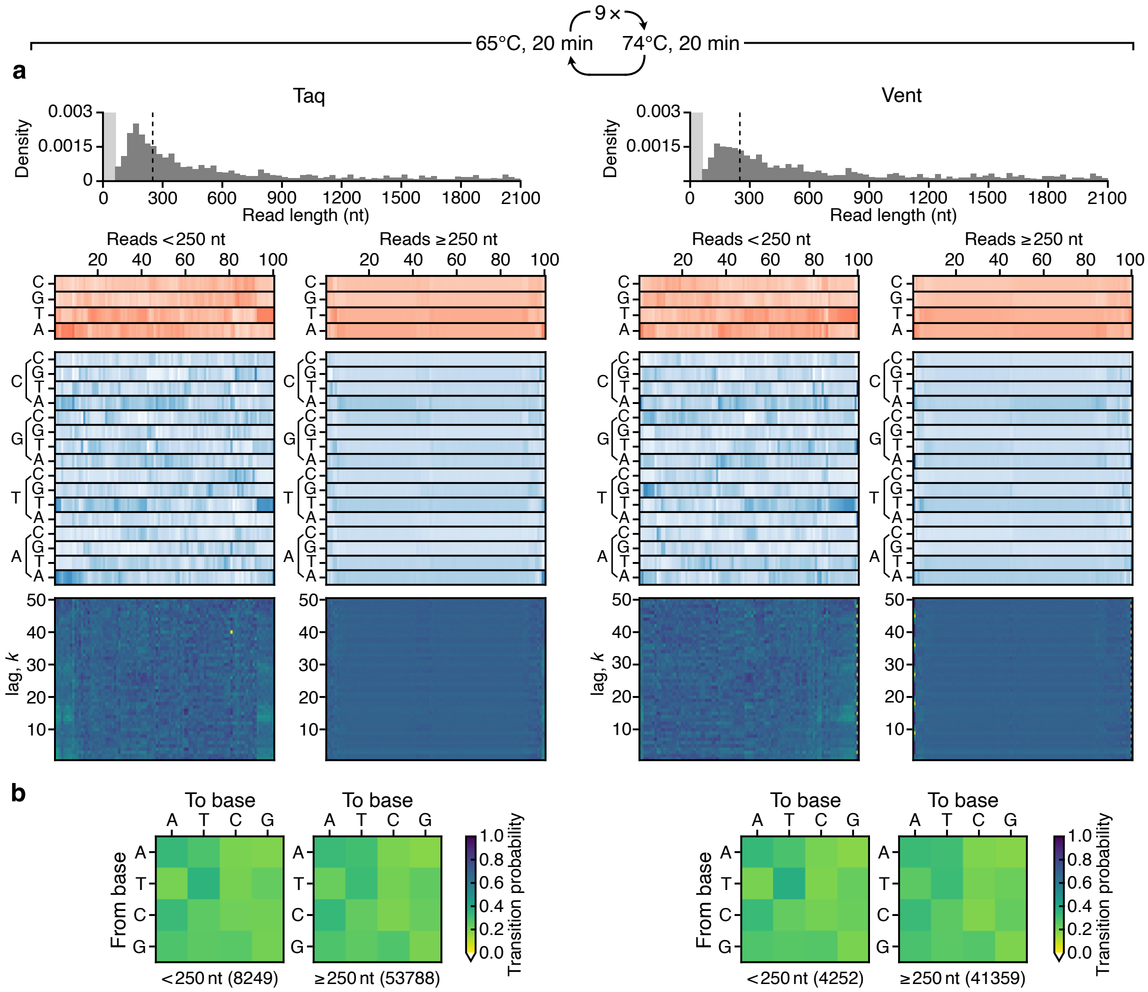

Supplementary Figure 10: Single-molecule analysis of the effect of thermocycling on doodling activity of Taq and Vent polymerases. (**a**) For each DNA polymerase (Taq, left; Vent, right), the top histogram shows the sequence length distribution with the lightly shaded region denoting the 0–65 nt range and dashed line denoting the 250 nt read length. Below this, heatmaps show for a random subset of reads smaller and larger than 250 nt (left and right plots, respectively) the following information (top–bottom): 1. Sequence composition (red heatmap), 2. Probability of transitioning from one base to another (blue heatmap), and 3. Autocorrelation analysis capturing the similarity of the sequence compared to itself after varying nucleotide shifts/lag *k* (blue to yellow heatmap. Reads are displayed vertically and hierarchically clustered such that similar sequences are grouped. (**b**) Base to base transition probabilities, separated by polymerase (Taq: left, Vent: right) and grouped by read length. Number in parenthesis denotes number of reads used to generate the matrix.

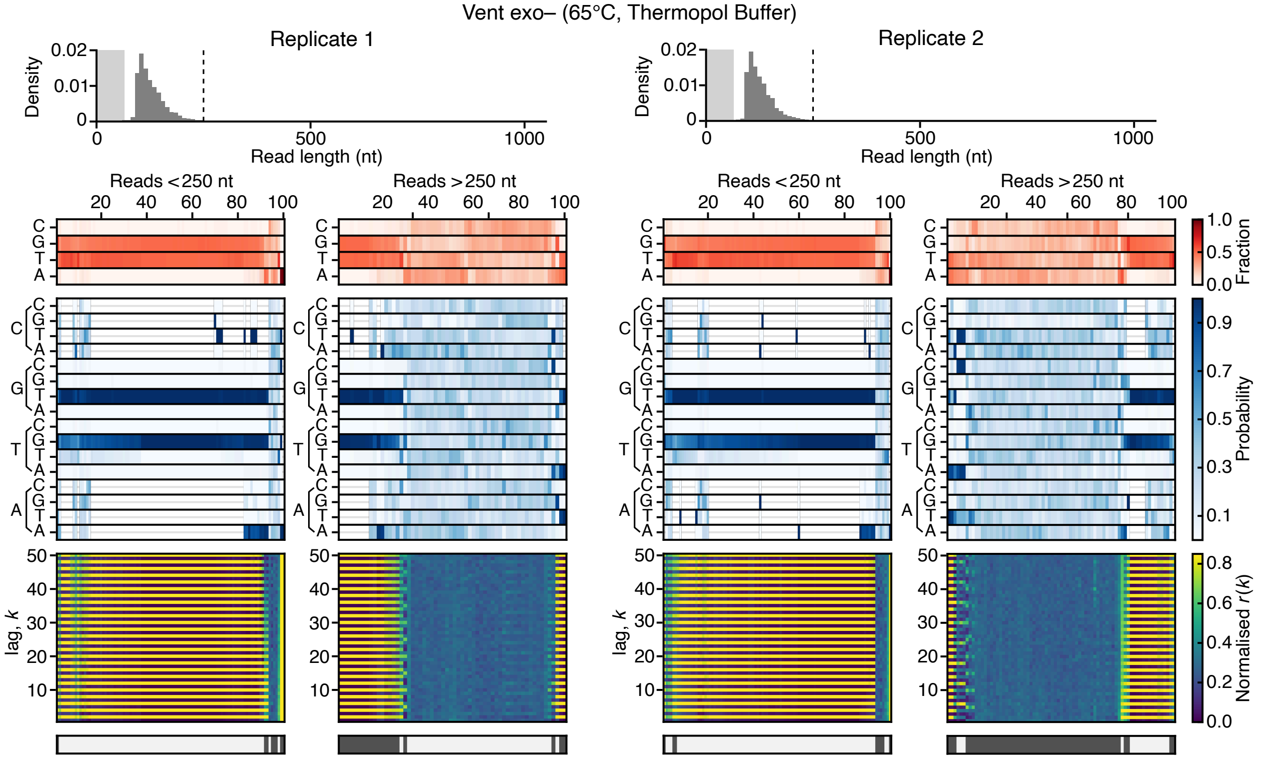

Supplementary Figure 11: Sequence analysis of Vent exo– (65°C, Thermopol Buffer). The top histograms show the sequence length distribution with the lightly shaded region denoting the 0–65 nt range and dashed line denoting the 250 nt read length. Below this, heatmaps show for a random subset of reads smaller and larger than 250 nt (left and right plots, respectively) the following information (top–bottom): 1. Sequence composition (red heatmap), 2. Probability of transitioning from one base to another (blue heatmap), 3. Autocorrelation analysis capturing the similarity of the sequence compared to itself after varying nucleotide shifts/lag *k* (blue to yellow heatmap), and 4. the seven top clusters of reads (alternating dark and light grey). Reads are displayed vertically and hierarchically clustered such that similar sequences are grouped.

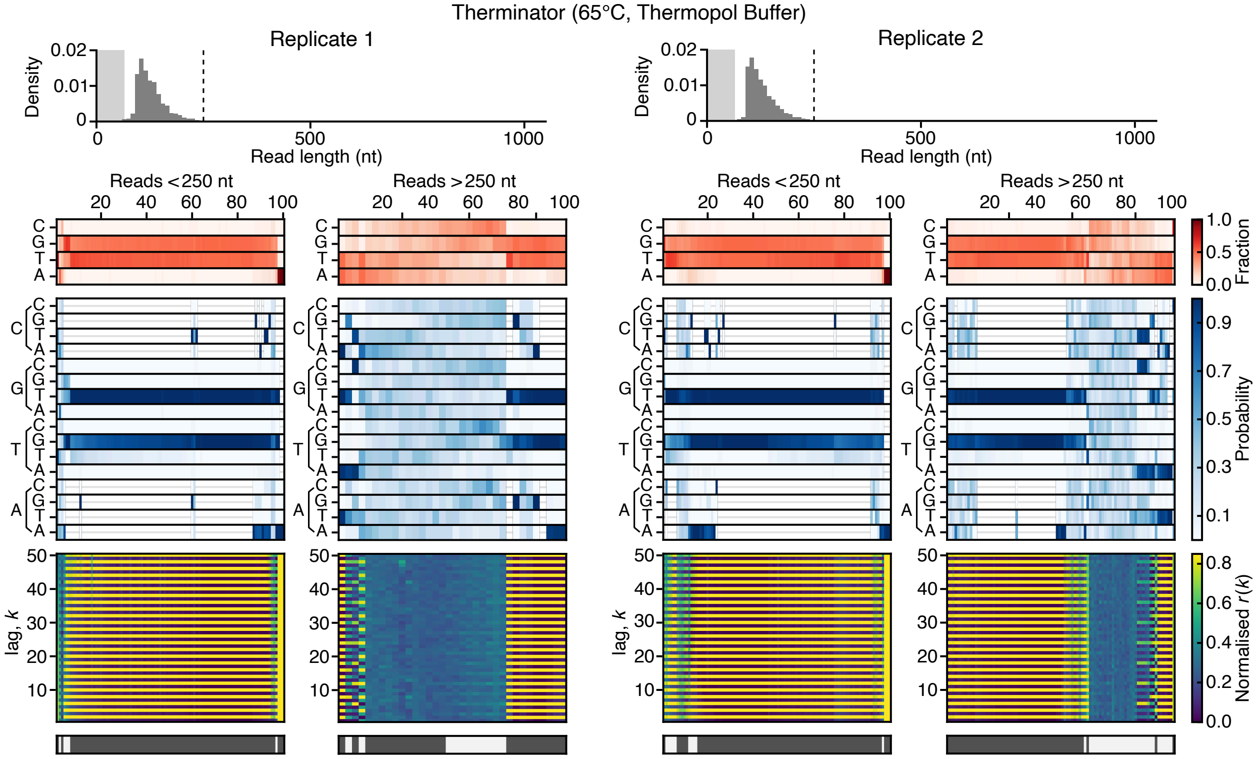

Supplementary Figure 12: Sequence analysis of Therminator (65°C, Thermopol Buffer). The top histograms show the sequence length distribution with the lightly shaded region denoting the 0–65 nt range and dashed line denoting the 250 nt read length. Below this, heatmaps show for a random subset of reads smaller and larger than 250 nt (left and right plots, respectively) the following information (top–bottom): 1. Sequence composition (red heatmap), 2. Probability of transitioning from one base to another (blue heatmap), 3. Autocorrelation analysis capturing the similarity of the sequence compared to itself after varying nucleotide shifts/lag *k* (blue to yellow heatmap), and 4. the seven top clusters of reads (alternating dark and light grey). Reads are displayed vertically and hierarchically clustered such that similar sequences are grouped.

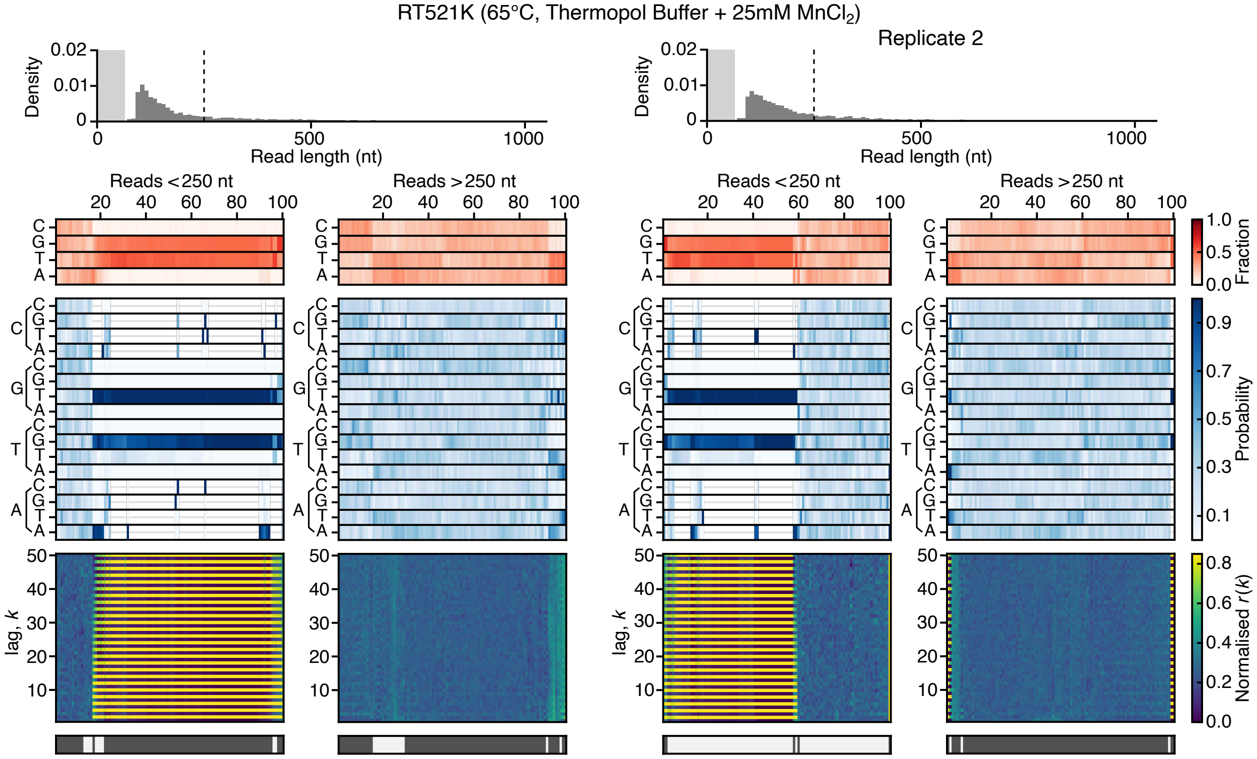

Supplementary Figure 13: Sequence analysis of RT521K (65°C, Thermopol Buffer, 25 mM $MnCl_2$). The top histograms show the sequence length distribution with the lightly shaded region denoting the 0–65 nt range and dashed line denoting the 250 nt read length. Below this, heatmaps show for a random subset of reads smaller and larger than 250 nt (left and right plots, respectively) the following information (top–bottom): 1. Sequence composition (red heatmap), 2. Probability of transitioning from one base to another (blue heatmap), 3. Autocorrelation analysis capturing the similarity of the sequence compared to itself after varying nucleotide shifts/lag *k* (blue to yellow heatmap), and 4. the seven top clusters of reads (alternating dark and light grey). Reads are displayed vertically and hierarchically clustered such that similar sequences are grouped.

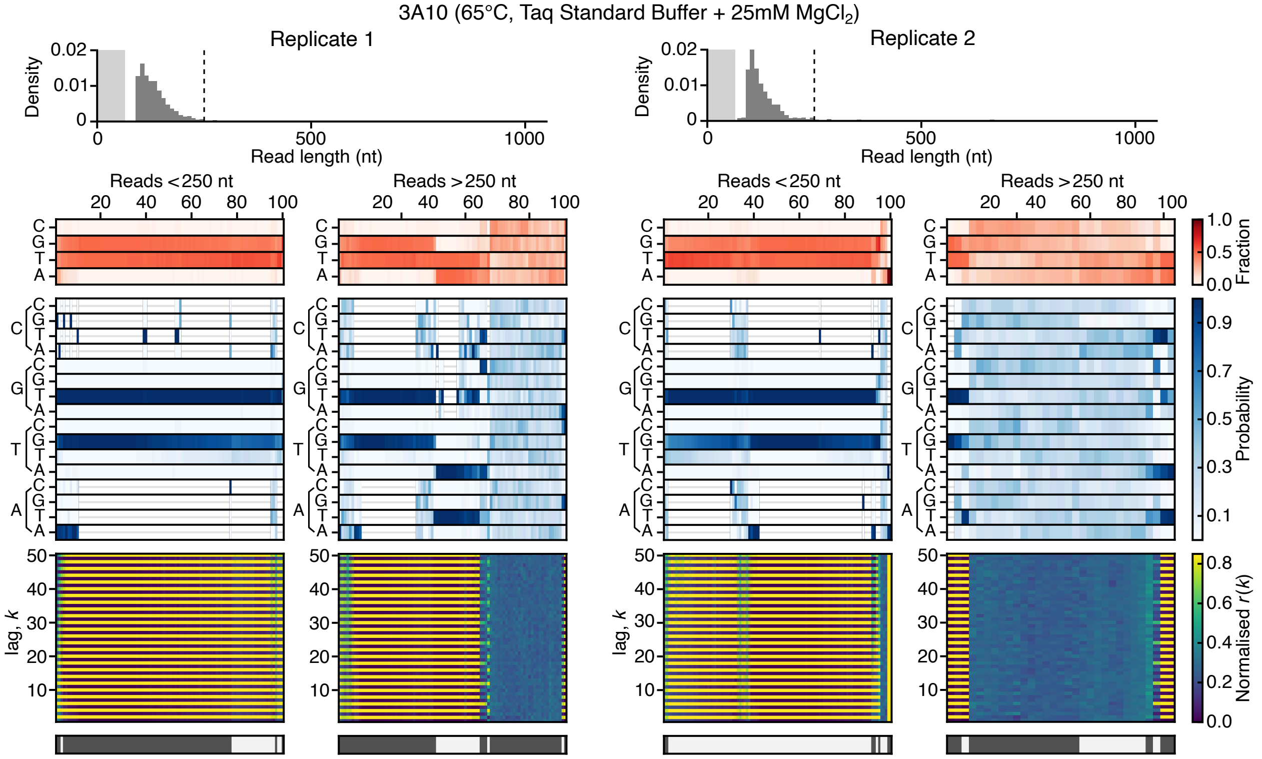

Supplementary Figure 14: Sequence analysis of 3A10 (65°C, Taq Standard Buffer, 25 mM $MgCl_2$). The top histograms show the sequence length distribution with the lightly shaded region denoting the 0–65 nt range and dashed line denoting the 250 nt read length. Below this, heatmaps show for a random subset of reads smaller and larger than 250 nt (left and right plots, respectively) the following information (top–bottom): 1. Sequence composition (red heatmap), 2. Probability of transitioning from one base to another (blue heatmap), 3. Autocorrelation analysis capturing the similarity of the sequence compared to itself after varying nucleotide shifts/lag *k* (blue to yellow heatmap), and 4. the seven top clusters of reads (alternating dark and light grey). Reads are displayed vertically and hierarchically clustered such that similar sequences are grouped.

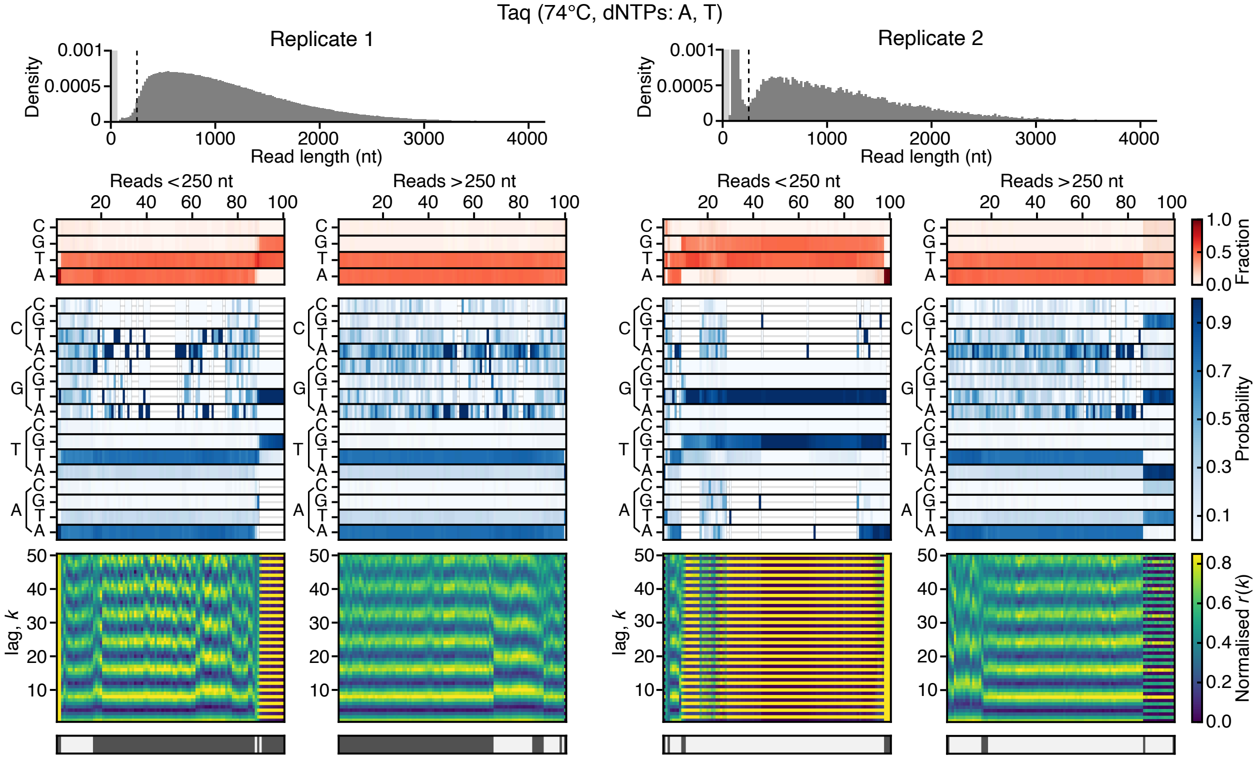

Supplementary Figure 15: Sequence analysis of Taq (65°C, Taq Standard Buffer, only adenine and thymine provided). The top histograms show the sequence length distribution with the lightly shaded region denoting the 0–65 nt range and dashed line denoting the 250 nt read length. Below this, heatmaps show for a random subset of reads smaller and larger than 250 nt (left and right plots, respectively) the following information (top–bottom): 1. Sequence composition (red heatmap), 2. Probability of transitioning from one base to another (blue heatmap), 3. Autocorrelation analysis capturing the similarity of the sequence compared to itself after varying nucleotide shifts/lag *k* (blue to yellow heatmap), and 4. the seven top clusters of reads (alternating dark and light grey). Reads are displayed vertically and hierarchically clustered such that similar sequences are grouped.

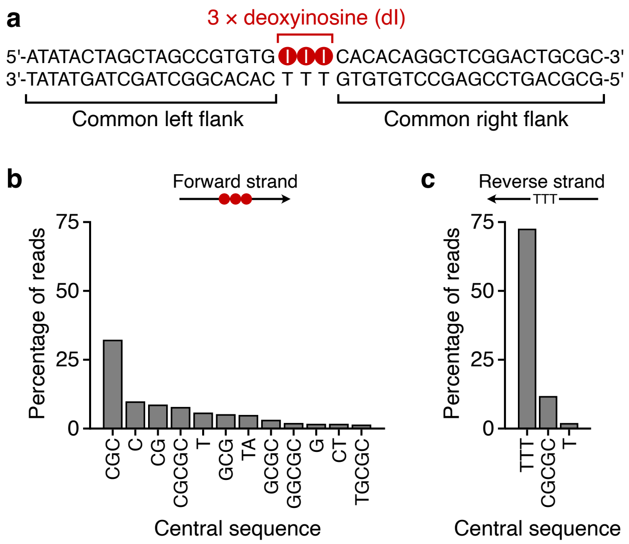

Supplementary Figure 16: Nanopore sequencing of inosine bases. Data from a single sequencing run. (**a**) Schematic of the synthesized ssDNA oligos that were nanopore sequenced containing complementary left and right flanks around a 3 bp region made up of inosine bases on one strand, and thymine on the other, to mimic deamination of adenine bases. (**b**) Basecalled nanopore sequencing data for the central region of the forward strand containing the three inosine bases. (**c**) Basecalled nanopore sequencing data for the central region of the reverse strand containing the three adenine bases.

Supplementary Table 1: Doodling activity of Taq with differing dNTP availability [a]

Polymerase	Temp. (°C)	Buffer	dNTPs	DNA mass [b] (ng/µL)	% Reads >250 nt	% Reads >1000 nt
Taq	74	Taq Standard	A	<4	48.6	11.8
				15	16.6	3.9
			T	<4	66.8	16.0
				11	37.0	10.8
			C	<4	45.5	10.8
				<4	27.7	7.2
			G	<4	42.7	10.7
				<4	23.2	6.3
			A, T	23	98.4	50.3
				<4	80.9	42.2
			A, C	12	63.6	17.1
				<4	31.8	8.3
			A, G	7	80.3	19.9
				<4	48.3	12.5
			T, C	8	59.0	14.2
				<4	22.4	5.3
			T, G	21	48.2	11.3
				<4	34.5	9.1
			C, G	19	73.6	17.3
				<4	30.5	7.3
			T, C, G	16	60.1	15.3
				<4	20.7	4.2
			A, T, C, G	100	97.9	24.4
				148	98.1	27.6

a. All reactions were 100 µL and run for 16 hours with DNA mass produced and read lengths given for both experimental replicates.

b. Values of <4 ng/µL were below the detection limit of the fluorometer used.
