# Supplementary material for: Analysis and control of untemplated DNA polymerase activity for guided synthesis of kilobase-scale DNA sequences
Source: Nat Commun. 2026 Feb 26;17:3251. doi: 10.1038/s41467-026-69915-x (PMC13061963; doi:10.1038/s41467-026-69915-x)
Supplement: Supplementary file 2 — Description of Additional Supplementary Files [file 41467_2026_69915_MOESM2_ESM.pdf]

Description of Additional Supplementary Files

File Name: Supplementary Data 1
Description: Contaminant sequences found in a subset of reads for the temperature switching reactions.

File Name: Supplementary Data 2
Description: Oligonucleotide sequences.
